# The Chemoprevention of Ovarian Cancer: the Need and the Options

**DOI:** 10.1007/s40495-018-0133-6

**Published:** 2018-05-02

**Authors:** Rishil J. Kathawala, Andrzej Kudelka, Basil Rigas

**Affiliations:** Department of Medicine, Stony Brook University, Stony Brook, NY USA

**Keywords:** Ovarian cancer, Chemoprevention, Oral contraceptives, PARP inhibitors, Tyrosine kinase inhibitors, NSAIDs

## Abstract

**Purpose of Review:**

Ovarian cancer (OvCa) is the most lethal of all gynecological cancers, with a 5-year survival around 46%, mainly due to limitations in early diagnosis and treatment. Consequently, the chemoprevention of OvCa emerges as an important option to control this dismal disease. Here, we discuss the role of risk assessment in the design of chemoprevention strategies for OvCa, describe candidate agents, and assess future directions in this field.

**Recent Findings:**

OvCa chemoprevention represents an opportunity for all women, especially those at high risk such as carriers of *BRCA1* or *BRCA2* mutations. The use of oral contraceptives confers substantial protection against OvCa including women at high risk, which increases with longer use. Despite strong evidence for their efficacy, safety concerns and the magnitude of the requisite interventional clinical trials seem to have precluded definitive studies of oral contraceptives for this application. Several other classes of drugs, including non-steroidal anti-inflammatory drugs, retinoids, angiopreventive agents, poly(ADP-ribose) polymerase inhibitors, and tyrosine kinase inhibitors have shown promise for OvCa chemoprevention.

**Summary:**

Currently, no agent is proven by interventional trials to possess chemopreventive properties against OvCa. The key opportunities in the chemoprevention of OvCa include the development of surrogate biomarkers for OvCa, the molecular definition of OvCa risk that will help select those who may benefit the most from chemoprevention, the identification of additional agents likely driven by understanding the molecular pathogenesis of OvCa, and the development of dedicated resources and support mechanisms for OvCa. Overall, there is significant optimism for the future of OvCa chemoprevention.

## Introduction

Ovarian cancer (OvCa) is the most lethal gynecologic malignancy with fewer than half of the patients surviving 5 years past diagnosis. The cost of this debilitating and deadly disease is great, both in terms of human suffering and of the financial burden to society, which is estimated at 5.7 billion annually in the USA alone [1].

In 2017, there will be approximately 22,440 new cases of OvCa in the USA leading to 14,080 deaths [2]. A woman’s lifetime risk of developing OvCa is 1 in 75, and her chance of dying of the disease is 1 in 100. The disease typically presents at late stages when the 5-year relative survival rate is only 29%; only 15% of patients are diagnosed with localized tumors (stage I) when the 5-year survival rate is 92% [3].

The combination of platinum/taxane chemotherapy and cytoreductive surgery represents the first-line treatment for OvCa. Most patients initially respond to this treatment. Unfortunately, most patients eventually develop resistance to chemotherapy. The patients whose tumor relapses within 6 months have a poor response to second-line treatment, with response rates of 7–12% [4]. Treatment with bevacizumab, a vascular endothelial growth factor (VEGF) inhibitor, pegylated liposomal doxorubicin, gemcitabine, or topotecan, either alone or in combination with platinum/taxane, has not been effective [5–9]. These patients are often not only resistant to platinum and taxane but also to other cytotoxic therapies (multidrug resistance) [10]. Disappointingly, novel-targeted agents like poly-ADP-ribose polymerase (PARP) inhibitors and OvCa screening strategies have not yet elicited satisfactory cure rates [11, 12].

The poor performance of therapeutic approaches has provided the impetus for the prevention of OvCa, a viable alternative to chemotherapy. Cancer chemoprevention is defined as the administration of a synthetic, natural, or biological agent at a safe dose to reduce or delay the development of malignancy or its recurrence [13]. Several agents have been investigated for their ability to prevent various cancers. The ideal chemopreventive agent should have little or no side effects, be efficacious, easy to administer, readily available, and cost-effective. Depending upon the risk and stage of carcinogenesis, chemoprevention is classified as primary, secondary, or tertiary [14]. *Primary chemoprevention* aims at preventing the development of premalignant lesions (often assessed by appropriate markers) and subsequent cancer in high-risk cohorts of the population. *Secondary chemoprevention* prevents the evolution of premalignant markers/lesions into cancer. Finally, *tertiary chemoprevention* prevents the recurrence of cancer. The validity of this approach has been successfully demonstrated in the breast, prostate, and colon cancer [15–17]. Thus, a chemopreventive strategy for the control of OvCa is a realistic option, especially for those at high risk.

Several agents have been evaluated for their ability to prevent OvCa, driven by epidemiological findings and to a lesser degree by our understanding of the biology of OvCa. With the exception of oral contraceptives, the quality of the data and the strength of the conclusions regarding candidate agents are in a state of evolution. Thus, chemoprevention for OvCa represents a research challenge and an opportunity to impact in a major way one of the most lethal human cancers. Here, we discuss the role of risk assessment in the design of chemoprevention strategies for OvCa, describe promising chemopreventive agents, and contemplate future directions in this field.

## Ovarian Cancer Risk as an Aid to Chemoprevention Strategies

In theory, every female should be given the opportunity of chemoprevention against OvCa. However, even if the ideal chemopreventive agent were to be available, logistical and financial considerations temper such a sweeping recommendation. Thus, identifying subgroups where the returns on the “chemoprevention investment” would be highest is a critical component in designing a realistic strategy.

Recent progress in the molecular genetics of OvCa has made risk stratification possible. OvCa has a distinctive biology and clinical course, reflecting the consequences of germline or somatic DNA changes (altered expression or function of proteins). The design of chemopreventive strategies against OvCa is informed by the epidemiology of OvCa, the mechanism of action of potential therapeutic agents, and the biology of ovarian carcinogenesis.

Women with germline mutations in OvCa predisposition genes, best defined in *BRCA1* and *BRCA2*, have a significant lifetime risk of developing ovarian, fallopian tube, or primary peritoneal carcinoma [18]. Depending on the mutations, the risk may be as high as 90% during their lifetime. Consequently, prophylactic or risk-reducing bilateral salpingo-oophorectomy may lower the risk by 80% for ovarian or fallopian tube cancers. While primary peritoneal cancer may still develop, breast cancer incidence can also be reduced by 50% [19]. Generally, in high-risk populations, it is recommended that bilateral salpingo-oophorectomy be performed by the age of 35 to 40 years or upon completion of childbearing [18, 19]. As such, women at high risk of developing these cancers are ideal candidates for screening, primary prevention strategies, or both. Given that efforts to define effective screening tools to improve overall survival are underway, primary prevention trials would be the best proposition in high-risk populations.

The strongest risk factor for OvCa is a family history of the disease, which is present in 10–15% of women [20]. There is a greater risk with a sporadic case of the disease in the family, but the risk is significantly increased with a hereditary cancer syndrome. For instance, women with a single family member affected by OvCa have a 4–5% risk of developing the disease, while in those with two affected relatives this risk is 7% [21•]. Women having at least two first-degree relatives with OvCa have a lifetime probability of 13–50% to develop OvCa [22].

Women with *BRCA* gene mutations are at significantly increased risk of OvCa, estimated to be 35–46% for *BRCA1* and 13–23% for *BRCA2* mutation carriers [20]. Overall, *BRCA* mutations account for up to 90% of the total hereditary OvCa cases, with most of these tumors representing the invasive serous adenocarcinoma histotype as opposed to borderline or mucinous histotype when compared with non-*BRCA* age-matched controls (odds ratio (OR) = 1.84; 95% confidence interval (CI), 1.21–2.79) [21•]. Sadly, in both *BRCA* mutation carrier and non-carrier women, OvCa often presents at stages III or IV [23]. The stage, grade, and histology-adjusted 5-year all-cause mortality was 45% in *BRCA1* carriers versus 47% in non-carriers (hazard ratio (HR) = 0.73; 95% CI, 0.64–0.84) and 36 versus 47% for *BRCA2* carriers (HR = 0.49; 95% CI, 0.39–0.61). It appears that *BRCA2* carriers have a better prognosis than non-carriers [24].

Among other risk factors, infertility is an independent risk factor. Nulliparous women may harbor a higher risk of OvCa independent of their use of fertility drugs. A recent study offered no convincing evidence of increased risk of invasive ovarian tumors with fertility drug treatment, although the risk of borderline ovarian tumors in subfertile women treated with in vitro fertilization may be increased [25]. The risk of OvCa is modestly increased in women with endometriosis and estimated at 2.5% [26]. Endometriosis-associated OvCa appears to occur in younger and nulliparous patients. These tumors are well-differentiated low-stage carcinomas that have a higher survival rate. There is a small increased risk associated with the polycystic ovary syndrome (OR = 2.52; 95% CI, 1.08–5.89) [27].

Obesity has been associated with ovarian cancer risk, but studies have yielded inconsistent findings. In one study, a small but statistically significant association was found between obesity and OvCa (OR = 1.3; 95% CI, 1.1–1.5) [28]. In a recent systematic review, 14 of 43 studies had a statistically significant positive association between ovarian cancer risk and higher body mass index, 26 studies found no significant association, and three found a negative association between ovarian cancer risk and higher body mass index [29]. These authors concluded that there is limited, inconsistent evidence of a positive association between obesity and OvCa risk.

Remarkably, a meta-analysis of 12 case-control studies indicates that hysterectomy is associated with 34% reduced risk of OvCa [30]. Another meta-analysis reported that women who had undergone tubal ligation had a 34% reduction in OvCa risk [31]. In addition, a case-control study by the Hereditary Ovarian Cancer Clinical Study Group found that tubal ligation lowered the rate of OvCa among *BRCA1* carriers by 60%, after adjustment for oral contraception use, parity, history of breast cancer, and ethnic group [32]. In a separate study, breastfeeding for a cumulative duration of more than 12 months was associated with decreased risk of epithelial OvCa compared with never breastfeeding (OR = 0.72; 95% CI, 0.54–0.97) [33].

The *Continuous Update Project* (CUP) will likely make a significant contribution to cancer risk stratification. The CUP analyzes global research on the effect of diet, nutrition, physical activity, and weight on cancer risk and survival including OvCa. Their findings are used to update cancer prevention recommendations, ensuring that everyone, from policymakers to members of the public, has access to the most up-to-date information on how to minimize the risk of developing the disease [34–37].

It is conceivable that a combination of genetic and lifestyle risk factors may define subsets of the population at low, intermediate, or high risk of OvCa to guide the selection of those who will benefit from the use of chemoprevention agents. In this context, there is a population-based program for OvCa risk prediction and stratification (*PROMISE* 2016 “Predicting Risk of Ovarian Malignancies, Improved Screening and Early detection”) [38]. This program develops and validates models for risk stratification, early detection, and diagnosis of OvCa, which incorporate clinical, epidemiological, proteomic, and genetic data. Thus, a constellation of new knowledge in various aspects of OvCa may help optimize our approach to OvCa chemoprevention.

## Chemopreventive Agents for Ovarian Cancer

Despite significant efforts, the achievement of optimal chemoprevention against OvCa remains an unmet need for the management of this recalcitrant clinical problem. The feasibility of chemoprevention of OvCa acquired excellent support (or, perhaps, “proof-of-concept”) from the seminal observations that oral contraceptives may prevent this dismal cancer [39, 40]. Congruent data show that oral contraceptive use reduces the risk of OvCa by over 20% for every 5 years a woman reports taking oral contraceptives [41••].

**Fig. 1:**
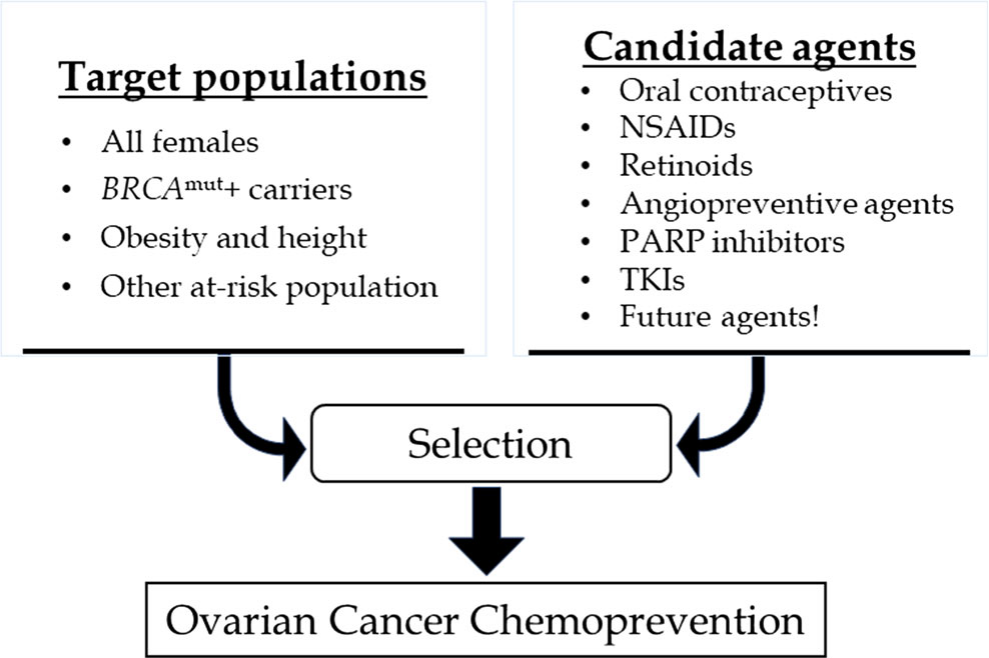
The chemoprevention of ovarian cancer. A match between an agent and its appropriate risk group will be essential for ovarian cancer chemoprevention. NSAIDs, non-steroidal anti-inflammatory drugs; PARP, poly (ADP-ribose) polymerase; TKIs, tyrosine kinase inhibitors

This effect of oral contraceptives is hypothesized to originate from their ability to suppress ovulation (the more ovulatory cycles a woman has, the higher her risk of developing OvCa after menopause) and from the ability of the progestins in oral contraceptives to eliminate premalignant cells. The latter has stimulated efforts to modulate molecular targets critical to ovarian carcinogenesis, providing a strong driver for this field. Below, we review the most promising agents (Fig. 1) and report on their current status.

### Oral Contraceptives

Oral contraceptives have been the most widely studied chemopreventive agents in OvCa. According to one study, ever use of oral contraceptives is associated with a 30% reduction in OvCa incidence in the general population, with greater risk reductions occurring with longer duration of use [42]. A large meta-analysis study that reviewed 55 studies relevant to OvCa outcomes showed that OvCa incidence was significantly reduced in oral contraceptive users (OR = 0.73; 95% CI, 0.66–0.81), and the magnitude of reduction increased with the duration of use [39], suggesting a strong duration-response relationship.

The risk reduction in OvCa by oral contraceptives has been observed in high-risk patients as well. In *BRCA1/2* mutation carriers, the use of oral contraceptives was associated with 20% risk reduction for up to 3 years of use and up to 60% for 6 or more years of use [43]. Looking specifically at the duration of use, each 10-year period of oral contraceptive use resulted in 36% relative risk reduction in the development of OvCa in *BRCA1/2* carriers [44]. A recent meta-analysis showed a significant risk reduction of OvCa in *BRCA1/2* mutation carriers on oral contraceptives (OR = 0.57; 95% CI, 0.47–0.70; *p* < 0.001) [45].

Additional supportive evidence for the use of oral contraceptives for the prevention of OvCa comes from a recent retrospective cohort study, which concluded that the use of oral contraceptives given prior to the diagnosis of OvCa was associated with better overall and progression-free survival. Specifically, the study reported that oral contraceptive use (ever versus never) was associated with better overall survival (HR = 0.73; 95% CI, 0.62–0.86); *p* = 0.0002) and better progression-free survival (HR = 0.71; 95% CI, 0.61–0.83); *p* < 0.0001) [41].

Over the years, a broad mechanistic network has been offered to explain the chemopreventive action of oral contraceptives in OvCa. Nearly all of the mechanisms proposed for ovarian carcinogenesis have been considered a target of oral contraceptives. First, it is hypothesized that repeated DNA damage during ovulation and its deficient recognition and repair are crucial to ovarian carcinogenesis. Consequently, the inhibition of ovulation by oral contraceptives may explain, at least in part, their chemopreventive effect against OvCa [46]. Second, the gonadotrophin hypothesis states that malignant transformation can be caused by the exposure of ovarian surface epithelium to excessive gonadotrophin levels [47]. Third, the role of progesterone has been the subject of mechanistic studies. For example, progesterone upregulated the expression of the tumor suppressor gene *p53* and inhibited the proliferation of sheep ovarian epithelial cells in vitro [48]. In addition, progesterone induced apoptosis in normal and malignant human ovarian epithelial cell lines and inhibited the proliferation of ovarian epithelial cell cultures from premenopausal and post-menopausal women [49]. Moreover, in a 3-year randomized controlled trial in non-human primates, the synthetic progestin levonorgestrel induced apoptosis in the ovarian surface epithelium [50]. Thus, it was speculated that exposure to high progesterone levels in pregnancy or progestins contained in oral contraceptives may induce apoptosis of cells in the ovarian surface epithelium.

Ovulation is a natural inflammatory process, the suppression of which by pregnancy, breastfeeding, or oral contraception reduces OvCa risk. During ovulation, ovarian surface epithelium cells are exposed to inflammatory mediators capable of inducing genetic changes that predispose to malignancy [51, 52]. Timely resolution of an inflammatory condition by oral contraceptives or anti-inflammatory agents is essential to prevent tumorigenesis emanating from the ovarian surface epithelium.

Because oral contraceptives have been shown to modestly increase the risk of breast cancer in the general population, there is concern regarding *BRCA* carriers [53, 54]. However, there is contrasting evidence to this notion. Progesterone has been reported to be growth-promoting, neutral, or anti-proliferative in breast cells, whereas synthetic progestins (especially the combination of conjugated equine estrogens and medroxyprogesterone acetate) are growth-promoting [55]. In contrast to progestins, progesterone in combination with estrogen has not been associated with increased risk of breast cancer [56]. In one study, no such association was found in women using contraceptive formulations with reduced estrogen concentrations or in the first 10 years following discontinuation of their use [57]. In contrast, a recent meta-analysis demonstrated a (not statistically significant) trend towards increased risk of breast cancer under contraceptive use in both *BRCA1* (OR = 1.19; 95% CI, 0.92–1.55) and *BRCA2* carriers (OR = 1.21; 95% CI, 0.93–1.58) compared to the general population (OR≈1.08) [58•].

In large pooled analyses, the use of oral contraceptives has been associated with prevention of 200,000 cases of OvCa and 100,000 deaths from this malignancy over 20 years [59, 60]. Compelling as these data may be, they have not been followed by large-scale prevention trials akin to those for colon cancer [41••, 61]. The lack of discrete, early surrogate end-points for OvCa necessitates decades of follow-up, making such trials both costly and complex. In addition, safety concerns, such as the increased risk of thrombophlebitis and breast cancer, may also dampen enthusiasm for such studies. Thus, this promising lead appears almost abandoned and alternative approaches are being assiduously explored.

### Non-Steroidal Anti-Inflammatory Drugs (NSAIDs)

Targeting inflammatory markers with NSAIDs is an attractive proposition for cancer prevention. There is a plethora of data supporting the use of NSAIDs in preventing cancer in a number of organs, and such drugs have given positive results in human interventional studies [62–64]. Despite evidence about the role of analgesic drug use in the prevention of OvCa, the chemopreventive potential of NSAIDs is yet to be established. For instance, the use of aspirin resulted in a statistically significant decrease in the risk of serous OvCa but not of mucinous or other ovarian tumors (OR = 0.60; 95% CI, 0.36–1.00) [65]. Contrastingly, the Multiethnic Cohort Study did not find compelling evidence to support an association between the use of NSAIDs and the risk of ovarian and endometrial cancers in a multiethnic population [66]. A meta-analysis study on the association between NSAIDs use and OvCa risk revealed no association between aspirin or non-aspirin NSAID use and OvCa risk, based on a random-effects model or a fixed-effects model. Furthermore, the analysis did not show any strong association between the frequency or duration of non-aspirin NSAID use and OvCa. Another case-control study reported an inverse correlation between low-dose aspirin and the risk of OvCa (adjusted OR = 0.94; 95% CI, 0.85–1.05) [67••]. The strongest inverse associations with low-dose aspirin use were seen for histological tumors representing the mucinous and endometrioid phenotypes. A provocative recent mechanistic study argued in favor of aspirin’s potential as an OvCa chemopreventive agent based on its ability to reverse the metabolic derangements caused by loss of *BRCA1*. Specifically, silencing *BRCA1* in ovarian surface epithelial and fallopian tube cells increased glycolysis accompanied by an increase in hexokinase-2, a key glycolytic enzyme. Aspirin counteracted the increase in hexokinase-2 and the increase in glycolysis induced by *BRCA1* impairment [68••]. Overall, these data indicate that there is no strong evidence of an association between aspirin/non-aspirin NSAID use and OvCa.

Acetaminophen is another analgesic drug reported to prevent OvCa. In one study, OvCa risk was significantly reduced in women receiving daily acetaminophen (OR = 0.52; 95% CI, 0.31–0.86) [69]. In the same study, there was a modest but non-significant inverse association between aspirin use and OvCa, but no association with ibuprofen use. A similar reduction in risk with daily acetaminophen use was also reported (adjusted OR = 0.56; 95% CI, 0.34–0.86) [70]. The benefits increased with increasing frequency and duration of use. Rodriguez et al. reported 45% lower death rate from OvCa in women using acetaminophen daily; however, this finding was not statistically significant. In this particular study, 5% of the women reported daily acetaminophen use and this small number of subjects could have contributed to a wider confidence interval [71].

Another target of chemopreventive interest is COX-2. COX-2 is an inducible enzyme of inflammation, catalyzing the early steps in the conversion of arachidonic acid to prostaglandins. However, it remains to be seen whether COX-2 inhibitors could prevent OvCa. In their exploratory study, Xin et al. showed that meloxicam (a selective COX-2 inhibitor) treatment decreased COX-2 expression in tumors obtained from OVCAR-3 xenografted mice by 2.5-fold compared with untreated tumors. Furthermore, meloxicam reduced microvessel density, induced apoptosis, and decreased prostaglandin E_2_ levels in serum as well as in ascites [72].

Taken together, the available data on the potential role of NSAIDs in OvCa present a rational argument for the continued study of these agents or improved versions of them [73, 74] in preclinical models and, as appropriate, in humans for OvCa chemoprevention.

### Retinoids

Retinoids, a class of compounds comprising vitamin A, its natural derivatives, and synthetic analogs, have been studied in both the prevention and treatment of gynecologic malignancies. Retinol and vitamin A derivatives influence cell differentiation, proliferation, and apoptosis and play an important role in a wide range of biological processes. Retinol is obtained from foods of animal origin. Retinol derivatives are fundamental for vision, while retinoic acid is essential for the skin and bone growth. Abnormal retinoid signaling has been identified as causative in certain cancers, including OvCa, making targeting retinoid pathways a therapeutic strategy [75••]. Indeed, retinoids are effective in the treatment of various malignancies and likely have a role in cancer prevention [75••, 76–78].

The intracellular retinoid concentration is regulated by a specific cellular retinol binding protein-1 (CRBP-1) [79•]. Downregulation or loss of CRBP-1 has been associated with stage I as well as stage II and III OvCa [79•]. A recent study reported that *CRBP-1*-transfected cells showed increased retinol-induced apoptosis, retinoid-induced reduced clonogenicity, and downregulation of proliferation and transcription of several genes, including *AKT1*, *AKT3*, *EGFR*, *FOS*, *JUN*, *STAT1*, and *STAT5A* [80]. These findings indicate that retinoids may have a role in OvCa prevention.

Fenretinide (N-(4-hydroxyphenyl)retinamide, 4-HPR), the most studied retinoid, demonstrated a significant cytotoxic effect in OvCa cell lines in vitro and in murine models through the induction of apoptotic and non-apoptotic cell death [81]. In a non-human primate model for the chemoprevention of OvCa, investigators evaluated the chemopreventive mechanism of action of fenretinide and oral contraceptives. While fenretinide alone enhanced apoptosis, the combination of fenretinide and oral contraceptives upregulated retinoid and estrogen receptors, providing a potential mechanism for their effect on the ovary [82]. In a clinical trial evaluating chemoprevention of OvCa with retinoids, patients with a history of breast cancer were treated with fenretinide. The incidence of OvCa decreased in these patients, but the effect ceased with treatment termination [83].

Collectively, these studies warrant further investigation of the chemopreventive properties of this class of compounds against OvCa.

### Natural Compounds

Several studies have demonstrated that plant-derived nutrients and nutraceuticals, such as flavonoids, flavones, and other antioxidants, can inhibit proliferation, induce apoptosis, and elicit cytotoxicity in cancer cells while sparing normal cells [84]. For example, curcumin, a polyphenolic natural compound, is beneficial in patients with platinum- or multidrug-resistant OvCa [85]. Withaferin A, a steroidal lactone, synergizes with doxorubicin against OvCa, which is brought about mainly by autophagy mediated by reactive oxygen species [86]. Withaferin-A, Amla extracts, ellagic acid, and resveratrol have been reported to prevent cisplatin resistance, while sulforaphane overcomes doxorubicin and cisplatin resistance in OvCa [87–90]. Curcumin, epigallocatechin 3-gallate, resveratrol, lycopene, sulforaphane, and Withaferin-A modulate pathways deregulated in cancer stem cells such as Wnt/β-catenin, Sonic hedgehog, and Notch, mainly interfering with the self-renewal of cancer stem cells [84, 91].

Overall, it appears that each of several natural compounds could prevent OvCa or be used in combination with other compounds, natural, or synthetic.

### Angiopreventive Agents

Tumor angiogenesis assists the growth of nascent tumors to clinically detectable masses. Thus, angiogenesis is an attractive target for therapy as well as prevention and is particularly appealing because these newly budding cells are relatively less transformed and thus less prone to develop resistance to therapeutics [92, 93••]. This concept has proven highly efficacious in preventing tumor growth in several animal models [94, 95]. By extension, angioprevention of OvCa might be achieved in women using appropriate agents.

Several anti-angiogenic drugs, including monoclonal antibodies and tyrosine kinase inhibitors, have been approved over the past 10 years for the treatment of OvCa [96]. In a recent study, angiopoietin-1 (Ang-1) and Tie2 levels were predictive biomarkers of the response to the VEGF-inhibitor bevacizumab in combination with carboplatin/paclitaxel in patients with advanced-stage/high-risk OvCa [97]. A retrospective analysis of the phase III GOG-0218 trial identified CD31 expression as a biomarker of improved progression-free survival and overall survival of patients with advanced OvCa treated with bevacizumab plus chemotherapy [98•].

The in vitro anti-angiogenic effect of retinoids is well studied. For instance, fenretinide was effective in blocking the migration of the OVCAR-3 OvCa cell line. This effect was mediated by downregulation of c-Jun, a key transactivator of genes involved in tumor progression and invasion, such as MMP-1 and MMP-3 [99, 100]. In another OvCa cell line, A2780, fenretinide induced c-fos and stimulated AP-1 transcriptional activity that was related to the induction of cell death via ceramide [101]. Recently, fenretinide was shown to inhibit OvCa cell invasion by disrupting actin cytoskeleton fibers and increasing FAK phosphorylation, both involved in cell motility and adhesion [102]. Likewise, Luo et al. found that chaetoglobosin K, a natural cytochalasan compound from the fungus *Diplodia macrospora*, could be used for angioprevention in OvCa [103]. Chaetoglobosin K significantly inhibited the secretion of key angiogenesis mediators, including Akt, hypoxia-inducible factor 1α (HIF-1α), and VEGF from A2780/CP70 and OVCAR-3 OvCa cell lines in vitro and in mouse models [103]. Kaempferol, a natural flavonoid present in many fruits and vegetables, has also been shown to inhibit angiogenesis and VEGF expression in human OvCa cell lines through both HIF-dependent (Akt/HIF) and HIF-independent (ESRRA) pathways [104].

These findings set the stage for further exploration of angiopreventive agents in the general population as well as in high-risk women, especially those with *BRCA* mutations.

### Poly(ADP-Ribose) Polymerase (PARP) Inhibitors

PARP inhibitors are pharmacological inhibitors of the enzyme poly (ADP-ribose) polymerase, which participates in DNA repair responses. PARP inhibitors are developed for multiple indications including acute life-threatening diseases (stroke and myocardial infarction), long-term neurodegenerative diseases, and cancer [105, 106]. Cancers with defective DNA repair mechanisms are more dependent on PARP than normal cells, making PARP an attractive therapeutic target. In particular, PARP inhibitors have been used to selectively inhibit cancers with *BRCA1/2* mutations. For example, single-agent PARP inhibitors have demonstrated durable anti-tumor efficacy in *BRCA*-mutated advanced OvCa in both its treatment and maintenance [107••, 108]. They include rucaparib (Rubraca), olaparib (Lynparza), and niraparib (Zejula). PARP inhibitors are also being evaluated in combination with chemotherapeutic and novel-targeted agents to potentiate anti-tumor activities [109–111].

Niraparib was evaluated as maintenance therapy in a multi-site phase III, double-blind, placebo-controlled study (ENGOT-OV16/NOVA trial) [112]. Treatment with niraparib reduced the risk of disease progression or death by 73% in patients with germline *BRCA* mutations (HR = 0.27) and by 55% in patients without germline *BRCA* mutations (HR = 0.45). In another phase III maintenance trial, the median progression-free survival was significantly greater in patients with *BRCA*-mutant carcinoma (16.6 months) in the rucaparib group versus patients in the placebo group (5.4 months; *p* < 0.0001) [113].

Even though in general, PARP inhibitors are well tolerated, further assessment of moderate and late-onset toxicity is required. With the advent of novel promising PARP inhibitors, it is highly likely that these agents might be useful as chemopreventive agents against OvCa. Semantics aside, delaying disease progression using PARP inhibitors, or other agents may prove to be a fruitful approach to control OvCa.

### Tyrosine Kinase Inhibitors (TKIs)

Tyrosine kinases are enzymes that activate various proteins in signal transduction cascades. Signaling pathways that are modulated by protein tyrosine kinases often play key roles in the initiation, progression, and metastasis of cancer cells. Consequently, tyrosine kinases have been evaluated as therapeutic targets.

Of the tyrosine kinases associated with OvCa, epidermal growth factor receptor (EGFR), Src, and Jak2 may have a significant role in its pathogenesis [114]. For instance, canertinib, a potent inhibitor of the EGFR kinase family is effective against OvCa. In OVCAR-5 and SKOV-3 OvCa 3D cell clusters and aggregates, canertinib significantly decreased cell growth and EGFR signaling proteins [115]. In a multicenter open-label phase II trial of OvCa patients who had failed platinum-based therapy, canertinib at a dose of 50 mg/day had favorable safety and tolerability [116]. Agents selective for HER-2 are also under development for OvCa. Numerous small-molecule TKIs targeting the VEGFR, PI3K-AKT-mTOR, MAPK, Src, PKC, and Wee1 signaling pathways are currently in clinical trials against OvCa [117].

TKIs could be of interest in OvCa prevention because of their efficacy and safety. In general, the dose-limiting toxicities for TKIs are skin rash and/or diarrhea. It is conceivable that selected TKIs could be used for the chemoprevention of OvCa, at least for subgroups of this disease.

## Conclusions

Primary and secondary prevention of OvCa plays an important yet suboptimal role in our effort to control cancer. The inherent limitations in biomarkers and imaging methods essentially dictate the need to pursue alternative approaches. It is in this context that the chemoprevention of OvCa becomes a compelling medical need. The fact that oral contraceptives confer protection from OvCa serves as a veritable proof-of-concept and legitimizes efforts to develop chemoprevention approaches for this disease.

As with every chemoprevention effort, the fundamental parameters to be addressed are the efficacy of the agent, its cumulative safety, and cost. Currently, no agent has been proven by interventional trials to possess chemopreventive properties against OvCa. Oral contraceptives are the closest to that designation, but as already mentioned, interventional trials evaluating their efficacy may not be a realistic expectation. At present, none of the other agents reviewed here seems promising enough in terms of both efficacy and safety to reasonably justify advancement to clinical testing.

Although this assessment appears bleak, there is no need to despair––yet! There are two reasons for optimism. First and foremost, molecular analyses of OvCa, not only are better defining the disease itself but also are promising objective risk assessment. Furthermore, it is reasonable to anticipate that in the not-too-distant future personalized chemoprevention based on the patient’s genotype will be a matter of medical routine. Second, our expanded understanding of the molecular pathogenesis of OvCa will likely lead to new agents, either specifically identified for chemoprevention or through repurposing of already available agents. The latter option often has a significant advantage in the extended safety data of such agents. To reiterate the dogma of chemoprevention, agent safety is paramount and stands in sharp contrast to what is acceptable for chemotherapeutic agents that are used in patients at imminent risk of death from cancer [118].

The research community focused on OvCa will have to address a plethora of specific issues, all in essence derivatives of the fundamental aspects of cancer chemoprevention mentioned above. For example, they will have to address innovative approaches to match agents and target subpopulations; design clinical trials such that the time to conclusions can be compressed, with strong surrogate markers being perhaps an integral part of this effort; define the minimum required period of intervention; and develop preclinical approaches for the selection of candidate agents. This list, without being exhaustive, underscores the magnitude of the challenge and the requirement for dedicated resources and support mechanisms, nicely exemplified by the *Continuous Update Project* (CUP).

It is unlikely that a single chemopreventive agent will be suitable for every woman at risk for OvCa. Moreover, it is now known that mutations other than *BRCA*, such as *BRIP1*, *RAD51C*, and *RAD51D*, also increase the risk of OvCa [119]. When considering best chemopreventive options, it is important for the patient and healthcare practitioner to have a clear sense of risk, as well as the potential benefits from, and side effects of, chemoprevention agents. A proper match between candidate agent and risk group would be essential for successful OvCa chemoprevention. In all likelihood, successful chemoprevention programs will include physicians with expertise in patient risk stratification and a firm understanding of agent pharmacology.

To use the old metaphor, the data reviewed here clearly indicate that the chemoprevention of OvCa is both a real and a paper tiger. Real in the sense that it is not only a formidable challenge that deserves our full effort but also a paper tiger since we are confident that the task at hand can be accomplished; and thus, we should not be discouraged by its current level of difficulty.
