# Correction: The Chemoprevention of Ovarian Cancer: the Need and the Options

**DOI:** 10.1007/s40495-018-0159-9

**Published:** 2018-09-10

**Authors:** Rishil J. Kathawala, Andrzej Kudelka, Basil Rigas

**Affiliations:** Department of Medicine, Stony Brook University, Stony Brook, NY USA

**Correction: Curr Pharmacol Rep (2018) 4(3):250–260**

10.1007/s40495-018-0133-6

The article The Chemoprevention of Ovarian Cancer: the Need and the Options, written by Rishil J. Kathawala, Andrzej Kudelka and Basil Rigas, was originally published electronically on the publisher’s internet portal (currently SpringerLink) on 2 May 2018 without open access.

With the author(s)’ decision to opt for Open Choice the copyright of the article changed on September 2018 to © The Author(s) 2018 and the article is forthwith distributed under the terms of the Creative Commons Attribution 4.0 International License (http://creativecommons.org/licenses/by/4.0/), which permits use, duplication, adaptation, distribution and reproduction in any medium or format, as long as you give appropriate credit to the original author(s) and the source, provide a link to the Creative Commons license and indicate if changes were made.

The original article has been corrected.

